# Metastatic Renal Cell Carcinoma with Level IV Thrombus: Contemporary Management with Complete Response to Neoadjuvant Targeted Therapy

**DOI:** 10.1155/2019/7102504

**Published:** 2019-03-14

**Authors:** Abhishek Bhat, Bruno Nahar, Vivek Venkatramani, Indraneel Banerjee, Oleksandr N. Kryvenko, Dipen J. Parekh

**Affiliations:** ^1^Department of Urology, University of Miami Miller School of Medicine, Miami, FL, USA; ^2^Department of Pathology and Laboratory Medicine, University of Miami Miller School of Medicine, Miami, FL, USA; ^3^Sylvester Comprehensive Cancer Center, University of Miami Miller School of Medicine, Miami, FL, USA

## Abstract

Renal cell carcinoma, particularly the most common clear cell type, is one of the most aggressive of urological cancers with significant risk of metastatic spread. It also has a propensity for venotropism with a proportion of tumors developing thrombi up to the right atrium. The response with newly adopted targeted therapy has been considered to be in the evolutionary stage with no clear role with respect to debulking or reducing the size of the inferior vena cava (IVC) thrombus. We describe a case of a right-sided metastatic RCC with Level IV thrombus initially managed with Pazopanib followed by Nivolumab and Adalimumab followed by cytoreductive nephrectomy and IVC thrombectomy in the post-targeted therapy setting with complete curative response.

## 1. Introduction

Renal cell cancers (RCC) comprise 3.8% of all new cancers in the United States with 90% of renal tumors being RCC and about 80% being clear cell type [1, 2]. The management of clinical Stage II and III renal cancers with inferior vena cava (IVC) extension includes radical nephrectomy [3]. Targeted therapy for advanced and metastatic RCC is widely used in first and second line treatments. We describe a case of advanced stage RCC with extension of thrombus into the right atrium and widespread visceral metastases who was initially treated with targeted therapy and then underwent posttherapy cytoreductive nephrectomy with IVC thrombectomy and metastasectomy with complete cure after downstaging had been achieved.

## 2. Case Report

**Figure 1:**
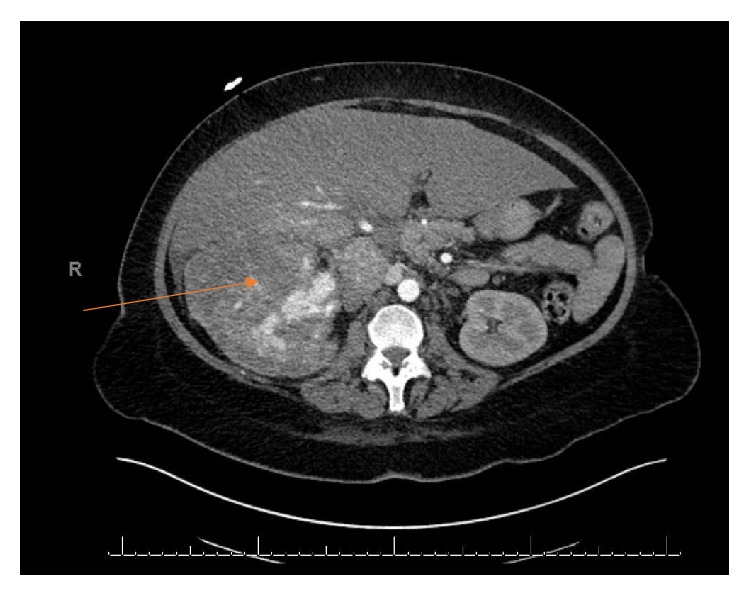
Right renal tumor with IVC thrombus.

**Figure 2:**
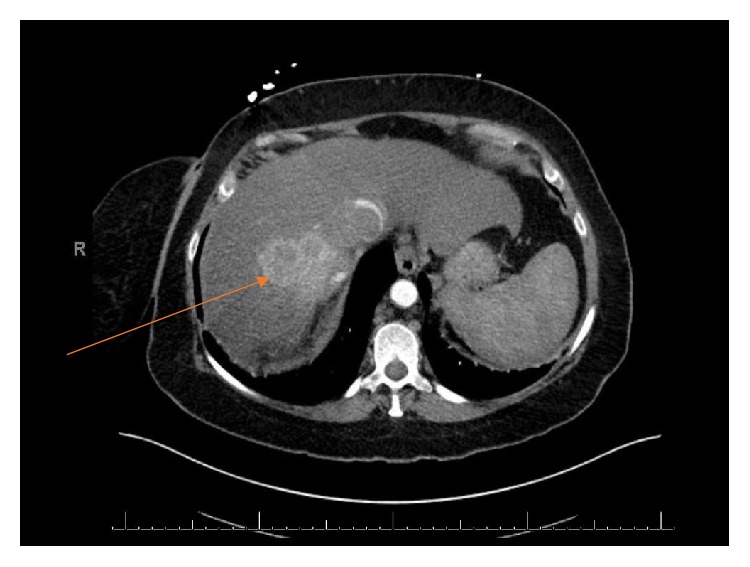
Liver metastases.

**Figure 3:**
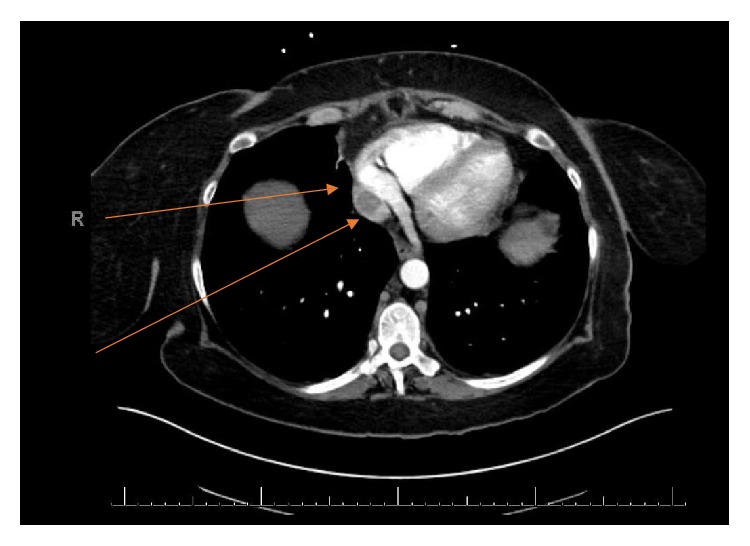
Extension of IVC thrombus to the right atrium.

A 57-year-old woman with good performance status was detected to have a large right renal mass with adrenal extension and tumor thrombus extending to the right atrium (Figures 1 and 3). She was also noted to have enhancing liver lesions (Figure 2), suggestive of metastatic disease (cT4N1M1). Pulmonary embolus and retroperitoneal lymphadenopathy were also noted. Biopsy of the mass revealed clear cell renal cell carcinoma, WHO ISUP nucleolar grade 3. She received Apixaban 10mg/day for management of pulmonary embolus.

She was started on Pazopanib 800 mg orally once daily which was later switched to Nivolumab after she developed upper gastrointestinal bleeding secondary to a duodenal ulcer.

Interval imaging 6 months after targeted therapy revealed a decrease in the size of the primary renal mass, although the thrombus extension into the IVC still persisted with development of new hilar lymphadenopathy and segmental pulmonary embolism. Nivolumab was continued and 3-month PET/CT showed further reduction in the size of the renal mass with thrombus extension now to the level of liver. There were new enhancing masses in the liver suspicious for metastases with subcarinal, precarinal, and bilateral hilar mediastinal lymphadenopathy.

**Figure 4:**
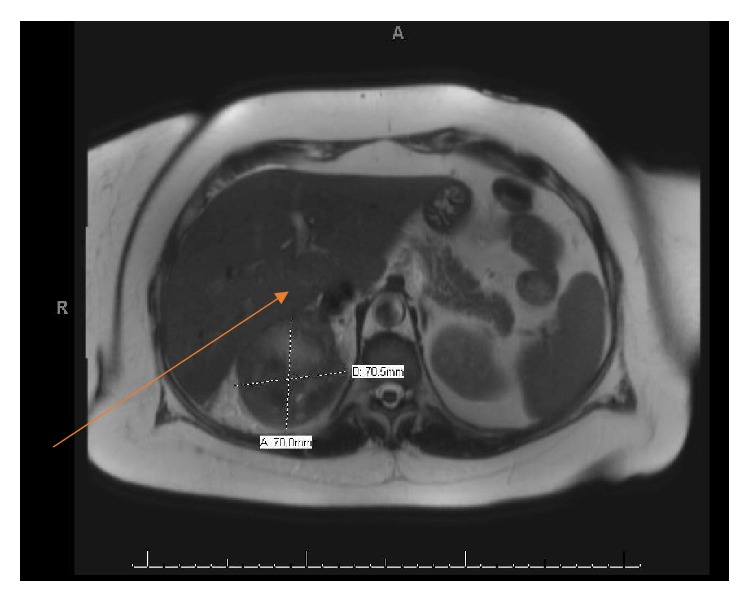
Resolution of liver metastases following neoadjuvant therapy.

**Figure 5:**
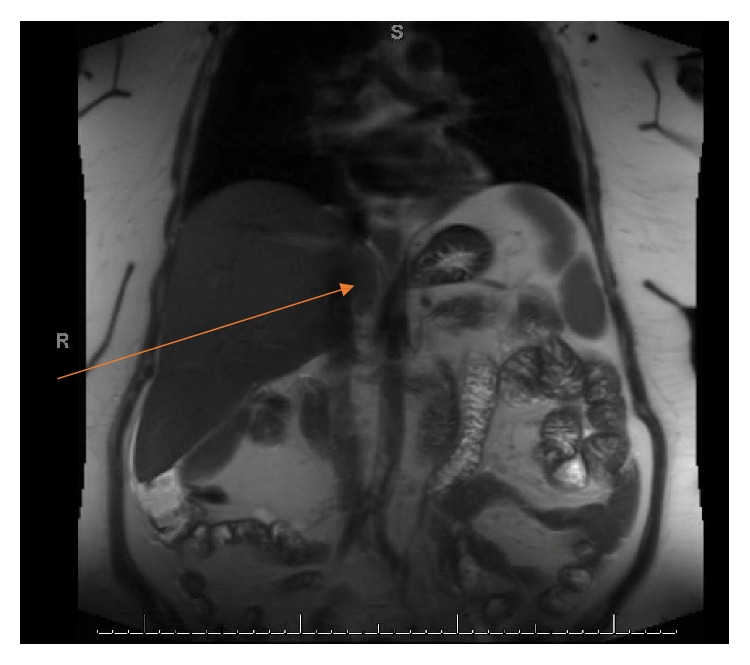
Presence of IVC thrombus at the infrahepatic level after neoadjuvant therapy.

She was initiated on Cabozantinib in addition to the Nivolumab in view of new lymphadenopathy. She tolerated the new regimen very well and was completely symptom-free with this therapeutic combination (Figure 4). New imaging with PET/CT showed no FDG avid lesions anywhere in the body including the IVC thrombus with significant reduction in the size of the renal mass. MRI Angiogram of the abdomen showed the IVC thrombus invading the IVC lateral wall and situated below the hepatic veins (Figure 5).

**Figure 6:**
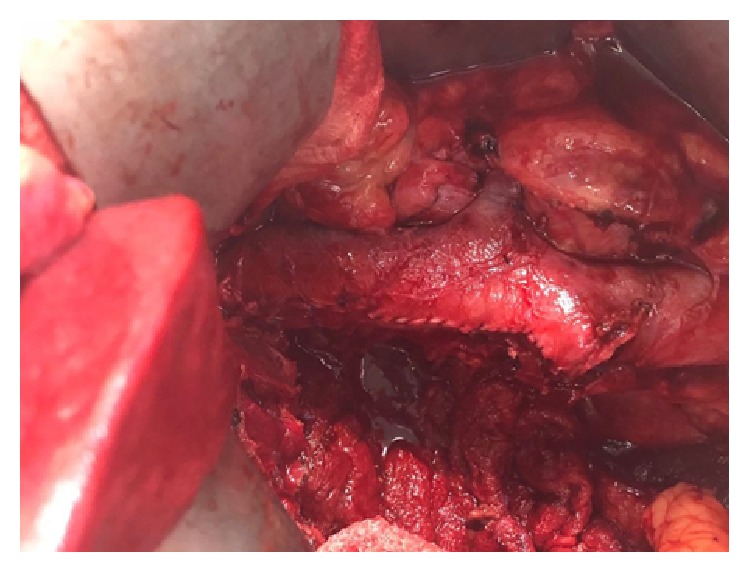
Radical Nephrectomy with complete IVC thrombectomy and caval reconstruction.

Based on the imaging, it was decided to proceed with posttherapy right cytoreductive nephrectomy with IVC thrombectomy. The anticoagulant was stopped and bridging was done with Heparin prior to the surgery. Intraoperative findings were that of a large renal mass with an infiltrating IVC thrombus in the retrohepatic location (using TEE assistance) with no gross evidence of metastases. Open radical nephrectomy with IVC thrombectomy and caval reconstruction was carried out with complete tumor thrombus resection (Figure 6). The patient did well postoperatively and was discharged on 6th postoperative day symptom-free.

**Figure 7:**
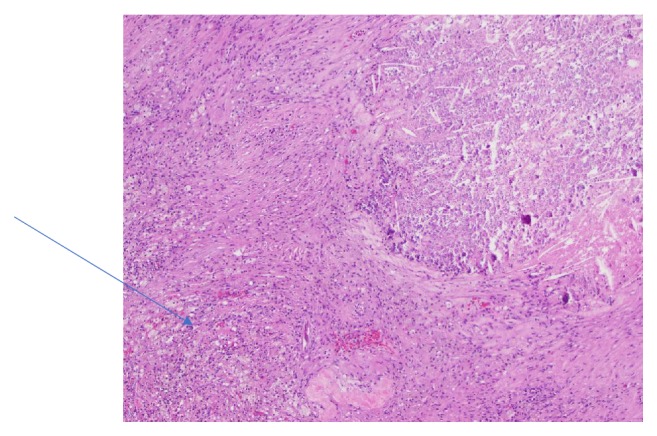
Necrotic tumor with calcifications surrounded by foamy histiocyte reaction.

**Figure 8:**
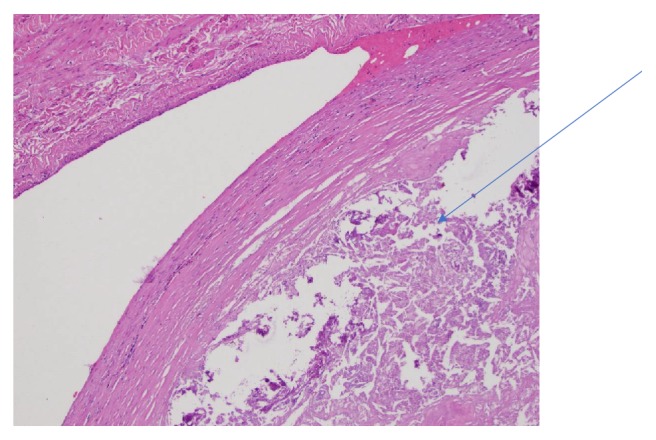
Necrotic tumor in renal vein.

Radical nephrectomy specimen examination showed a necrotic nonviable carcinoma consistent with an RCC with complete response measuring 7 cm in size with perinephric extension. The tumor was extensively sampled and no viable carcinoma was detected (Figure 7). The necrotic tumor was surrounded by a thick fibromuscular pseudocapsule typically see in clear cell renal cell carcinoma which maintains its structure and did not have necrosis [4]. IVC specimen showed a necrotic nonviable carcinoma in the lumen of renal vein as well (Figure 8). The AJCC 8th edition pathological stage was ypT0Nx Mn/a.

She was fully back to her activities of daily living with no complaints at the time of her first follow-up visit to the clinic at the postoperative visit at 3 weeks. She will continue to follow up with both the Urological Oncology and Medical Oncology teams. She has not been planned for adjuvant targeted therapy at this stage in view of no evidence of residual disease.

## 3. Discussion

The management of metastatic RCC with Level IV IVC thrombus is complex and requires multimodality treatments. Current available literature supports the potential increasing role of targeted therapy in the neoadjuvant setting for the management of metastatic RCC and advanced RCC. The therapy does seem safe and feasible with no significant complications. The priority in this case from our standpoint was to achieve substantial downgrading of the tumor and to ensure control of metastatic disease. When this had been accomplished, the next step was posttherapy cytoreductive nephrectomy with IVC thrombectomy.

Pazopanib is a preferred Category 1 option for first line treatment of patients with relapsed or medically unresectable Stage IV clear cell RCC. This is supported by the results of the COMPARZ and the PISCES trial which showed a comparable oncological control with fewer side effects reported [5–7].

The data for first line Nivolumab in combination with Ipilimumab for favorable risk patients has been mixed. The Checkmate 214 trial showed that the combination of Nivolumab and Ipilimumab produced a higher objective response rate (42% vs 27%, p<0.001) and a higher complete response rate (9% vs 1%, p<0.001) compared to Sunitinib monotherapy [7]. This has led to the combination being recommended as a Category 1, preferred treatment option for first line treatment for intermediate and poor risk patients with previously untreated, relapsed or medically unresectable predominantly Stage IV clear cell renal cell carcinoma.

Patients treated with Cabozantinib as a primary treatment modality showed a significantly increased median progression free survival (PFS) compared to those treated with Sunitinib. They also showed a significantly higher objective response rate (ORR) compared to Sunitinib (46% vs 18%) [8].

One of the benefits of preoperative systemic treatment is the possible reduction in the size of the primary tumor to make it resectable. This has received variable reporting in literature with wide variation in the treatment protocols and adoption. Despite this, there does seem to be some evidence to achieve downstaging in truly unresectable tumors. Thomas et al. reported a series of 19 patients with unresectable or bilateral tumors who received neoadjuvant Sunitinib. 15 patients were able to undergo radical nephrectomy thereafter with mean reduction in tumor size by 16% and significant reduction in tumor burden [9]. In another case series, there was significant tumor size reduction by 14%. Three of 10 patients underwent radical nephrectomy after 4 weeks of Sunitinib with significant tumor necrosis being reported on pathology due to the antiangiogenic action of the targeted therapy [10].

The commonly used RECIST criteria to look for response to standardized treatment may not be effective as antiangiogenic therapy may not affect the size of the tumor but effectively produces intratumoral necrosis which is not assessed at all [11]. Further, it has been demonstrated in studies that loss of intratumoral enhancement and density may be more reliable indicators of preoperative tumor therapy than tumor size alone [12].

Preoperative therapy to produce a reduction in the level of IVC thrombus has remained ineffective with IVC thrombectomy alone remaining the standard of care. Much of the existing literature on the use of preoperative therapy in the setting of an IVC thrombus comes from single center studies. The study by Cost et al. demonstrated there was minimal clinical effect on RCC thrombi with only one case of thrombus level regression from Level IV to Level II [13].

There have been data in the form of randomized controlled trials to demonstrate that overall survival does not increase with a cytoreductive nephrectomy in intermediate and poor risk patients. However this data is not robust for favorable risk patients [14]. Our patient was in the intermediate risk category in view of synchronous metastatic disease and targeted therapy along with posttherapy cytoreductive nephrectomy showed dramatic response, with downstaging of the tumor thrombus as well. This aspect of targeted therapy needs more studies and trials and could prove to be an effective management of select patients with advanced disease.

## 4. Conclusion

Downstaging the advanced primary tumor, tumor thrombus, and metastases provides an option of radical nephrectomy. Although current literature does not advocate neoadjuvant therapy for downstaging the level of IVC thrombus, we found a significant downstaging in the level of the thrombus from Level IV to Level II. Although the standards for neoadjuvant therapy have still not been established due to paucity of literature, more research is required to shed light on management of such tumors.

## References

[1] Leibovich B. C., Lohse C. M., Crispen P. L. (2010). Histological subtype is an independent predictor of outcome for patients with renal cell carcinoma. *The Journal of Urology*.

[2] Moch H., Gasser T., Amin M. B., Torhorst J., Sauter G., Mihatsch M. J. (2000). Prognostic utility of the recently recommended histologic classification and revised TNM staging system of renal cell carcinoma: a swiss experience with 588 tumors. *Cancer*.

[3] Luo J.-H., Zhou F.-J., Xie D. (2010). Analysis of long-term survival in patients with localized renal cell carcinoma: laparoscopic versus open radical nephrectomy. *World Journal of Urology*.

[4] Roquero L., Kryvenko O. N., Gupta N. S., Lee M. W. (2015). Characterization of fibromuscular pseudocapsule in renal cell carcinoma. *International Journal of Surgical Pathology*.

[5] Motzer R. J., Haas N. B., Donskov F. (2017). Randomized phase III trial of adjuvant pazopanib versus placebo after nephrectomy in patients with localized or locally advanced renal cell carcinoma. *Journal of Clinical Oncology*.

[6] Motzer R. J., Hutson T. E., Cella D. (2013). Pazopanib versus sunitinib in metastatic renal-cell carcinoma. *The New England Journal of Medicine*.

[7] Motzer R. J., Tannir N. M., McDermott D. F., Arén Frontera O., Melichar B., Choueiri T. K. (2018). Nivolumab plus ipilimumab versus sunitinib in advanced renal-cell carcinoma. *The New England Journal of Medicine*.

[8] Choueiri T. K., Halabi S., Sanford B. L. (2017). Cabozantinib versus sunitinib as initial targeted therapy for patients with metastatic renal cell carcinoma of poor or intermediate risk: the alliance A031203 CABOSUN trial. *Journal of Clinical Oncology: Official Journal of the American Society of Clinical Oncology*.

[9] Thomas A. A., Rini B. I., Lane B. R. (2009). Response of the primary tumor to neoadjuvant sunitinib in patients with advanced renal cell carcinoma. *The Journal of Urology*.

[10] Bex A., van der Veldt A. A. M., Blank C. (2009). Neoadjuvant sunitinib for surgically complex advanced renal cell cancer of doubtful resectability: Initial experience with downsizing to reconsider cytoreductive surgery. *World Journal of Urology*.

[11] Hellenthal N. J., Underwood W., Penetrante R. (2010). Prospective clinical trial of preoperative sunitinib in patients with renal cell carcinoma. *The Journal of Urology*.

[12] Cowey C. L., Fielding J. R., Kimryn Rathmell W. (2010). The loss of radiographic enhancement in primary renal cell carcinoma tumors following multitargeted receptor tyrosine kinase therapy is an additional indicator of response. *Urology*.

[13] Cost N., Delacroix S., Sleeper J. (2011). The impact of targeted molecular therapies on the level of renal cell carcinoma vena caval tumor thrombus. *European Urology*.

[14] Méjean A., Ravaud A., Thezenas S., Colas S., Beauval J-B., Bensalah K. (2018). Sunitinib alone or after nephrectomy in metastatic renal-cell carcinoma. *The New England Journal of Medicine*.

