# Corrigendum to “Metastatic Renal Cell Carcinoma with Level IV Thrombus: Contemporary Management with Complete Response to Neoadjuvant Targeted Therapy”

**DOI:** 10.1155/2019/5734970

**Published:** 2019-11-20

**Authors:** Abhishek Bhat, Bruno Nahar, Vivek Venkatramani, Indraneel Banerjee, Oleksandr N. Kryvenko, Dipen J. Parekh

**Affiliations:** ^1^Department of Urology, University of Miami Miller School of Medicine, Miami, FL, USA; ^2^Department of Pathology and Laboratory Medicine, University of Miami Miller School of Medicine, Miami, FL, USA; ^3^Sylvester Comprehensive Cancer Center, University of Miami Miller School of Medicine, Miami, FL, USA

In the article titled “Metastatic Renal Cell Carcinoma with Level IV Thrombus: Contemporary Management with Complete Response to Neoadjuvant Targeted Therapy” [1], the last sentence in the Abstract should be corrected from “We describe a case of a right-sided metastatic RCC with Level IV thrombus initially managed with Pazopanib followed by Nivolumab and Adalimumab followed by cytoreductive nephrectomy and IVC thrombectomy in the post-targeted therapy setting with complete curative response” to “We describe a case of a right-sided metastatic RCC with Level IV thrombus initially managed with neoadjuvant targeted therapy followed by cytoreductive nephrectomy and IVC thrombectomy in the post-targeted therapy setting with complete curative response”.
